# Prevalencia del déficit de vitamina D y su relación con la hormona paratiroidea

**DOI:** 10.1515/almed-2021-0093

**Published:** 2022-01-05

**Authors:** Alejandro José Ravelo Marrero, Carlos Antonio Guillén, Miriam Menacho Román, Marta Rosillo, José Manuel Del Rey, Ana Gómez, María Andreína Terán, Mónica Vázquez, Ignacio Arribas

**Affiliations:** Bioquímica Clínica, Hospital Ramón y Cajal, Madrid, España; Reumatología, Hospital Ramón y Cajal, Madrid, España

**Keywords:** 25-hidroxivitamina D, hormona paratiroidea, prevalencia

## Abstract

**Objetivos:**

Evaluamos la prevalencia del déficit de la 25-hidroxivitamina D (25-(OH)D) en nuestro entorno, en función de la estación del año, el sexo y la edad de los sujetos. Así mismo, analizamos su relación con los niveles de la hormona paratiroidea (PTH).

**Métodos:**

La población de estudio estaba compuesta por pacientes con peticiones de análisis de 25-(OH)D entre el 1 de enero y el 31 de diciembre de 2018, registradas en la base de datos del sistema informático del laboratorio. Se excluyeron las muestras de pacientes pediátricos (<18 años), así como de aquellos sujetos con factores que pudieran afectar a los niveles de 25-(OH)D y/o PTH (e.g. insuficiencia renal, enfermedad hepática, trastornos de la PTH).

**Resultados:**

En los 33.601 pacientes (24.028 mujeres y 9.573 hombres), la prevalencia del déficit de 25-(OH)D fue del 48%. Se observó una mayor prevalencia entre los hombres, frente a las mujeres (53% vs. 46%). Por grupos de edad, la prevalencia fue mayor en el cuartil 1 (Q1, 74–87 años) y menor en el cuartil 2 (Q2, 60–73 años). Por estación del año, este déficit fue mayor en primavera (diferencias no significativas con respecto al invierno) y menor en verano. La relación entre la 25-(OH)D y la PTH se evaluó en 9.368 personas. El análisis de regresión lineal mostró una asociación débil (coeficiente – 0,303). El análisis de regresión logística múltiple reveló una relación significativa entre el déficit de 25-(OH)D y niveles elevados de PTH (*Odds ratio* (OR), 1,63). Otros factores de riesgo asociados a mayores niveles de PTH fueron el sexo (OR, 1,27), la estación del año (invierno, OR 1,63, primavera OR 1,16) y la edad (cuartil 1, OR, 3).

**Conclusiones:**

La prevalencia del déficit de 25-(OH)D variaba según el sexo, la edad y la estación del año. Además, la elevación de la PTH está principalmente relacionada con niveles bajos de 25-(OH)D, el género femenino, la estación y la edad.

## Introducción

En los últimos años la deficiencia de vitamina D ha sido objeto de creciente preocupación entre la comunidad médica, que se ha visto reflejada en un aumento en las peticiones de pruebas diagnósticas y prescripción de suplementos [1]. No obstante, sigue sin existir consenso sobre la definición de la misma, y siguen publicándose artículos sobre este tema, como el de Cashman y col. [2]. Los umbrales que determinan el déficit se han establecido en concentraciones séricas de 25-hidroxivitamina D (25-(OH)D) entre <10 ng/mL y <30 ng/mL, dependiendo del país, la organización de salud y la sociedad científica [3].

Desde un punto de vista poblacional, el Instituto de Medicina de los EE.UU (IOM, por sus siglas en inglés) ha establecido una ingesta dietética recomendada de vitamina D. Tomando los datos sobre salud ósea como referencia, indican que una concentración sérica de 20 ng/mL satisfaría las necesidades del 97,5% de la población [4]. Del mismo modo, la Autoridad Europea de Seguridad Alimentaria ha establecido el mismo rango de referencia que el IOM, aunque reconocen que los datos actualmente disponibles no permiten determinar si dicho objetivo es alcanzable en la mitad o en la mayoría de la población [5]. Por otro lado, el Comité de Evaluación Científica de Nutrición del Reino Unido (UK Scientific Assessment Committee on Nutrition), ha establecido el umbral de referencia en <10 ng/mL (<25 nmol/L), basándose en el mayor riesgo de raquitismo en la población pediátrica y de osteomalacia en adultos [6].

Los comités de expertos de la Sociedad de Endocrinología (Endocrine Society), la Fundación Nacional de la Osteoporosis (National Osteoporosis Foundation), la Fundación Internacional de la Osteoporosis (International Osteoporosis Foundation), y Sociedad Americana de Geriatría (American Geriatric Society), partiendo de la perspectiva de la práctica clínica, recomiendan concentraciones séricas de 25-(OH)D >30 ng/mL (>75 nmol/L), especialmente en las personas de edad avanzada [7], [8], [9], [10], [11].

Las estimaciones sobre la prevalencia del déficit de 25-(OH)D vienen determinadas por factores como el sexo, la edad, la estación del año, la raza y la metodología (diseño del estudio, técnica empleada en la cuantificación de la 25-(OH)D, etc.), lo que origina grandes discrepancias entre estudios [3, 12], [13], [14], [15], [16]. Las estimaciones de prevalencia más recientes se basan en estudios nacionales, así como en la estandarización de la cuantificación de la 25-(OH)D por parte del Programa para la Estandarización de la Vitamina D (VDSP) llevado a cabo por los Institutos Nacionales de Salud de los EE.UU. De este modo, para un umbral del VDSP de 25-(OH)D <12 ng/mL, la prevalencia fue del 5,9% en EE.UU [17], 7,4% en Canadá [18], y 13% en Europa, alcanzando el 40% cuando el punto de corte se establece en <20 ng/mL [19].

También se ha intentado establecer un valor óptimo de 25-(OH)D, en función de su efecto sobre la hormona paratiroidea (PTH). Este valor se ha definido como la concentración mínima de 25-(OH)D necesaria para evitar hiperparatioridismo secundario y la osteoporosis que éste origina [20, 21]. Sin embargo, no ha sido posible establecer un valor consistente de 25-(OH)D, por debajo del cual se acaba desarrollando hiperparatioridismo. Una amplia variedad de estudios transversales han demostrado que los niveles de 25-(OH)D son inversamente proporcionales a los niveles de PTH, pero aportan resultados contradictorios en los valores de corte, y una prevalencia de niveles aumentados de PTH del 10–33% en los pacientes con hipovitaminosis D [22, 23].

Esta observación puede deberse a múltiples factores, como las diferencias en la estandarización de las pruebas de cuantificación de la PTH, y la naturaleza heterogénea de las poblaciones de estudio, en cuanto a raza, edad y género.

El Estudio Nacional Sobre Salud y Nutrición (NHANES) de 2003–2004 y 2005–2006 reveló diferencias raciales significativas en la relación entre los niveles de 25-(OH)D y de PTH superiores e inferiores al umbral que se aplica habitualmente para determinar el déficit de vitamina D (20 ng/mL), existiendo una relación inversamente proporcional entre los americanos blancos y los americanos mejicanos, pero no con los afroamericanos [24].

El objeto del presente estudio es evaluar la prevalencia del déficit de vitamina D en nuestro entorno en relación con la estación del año, sexo y edad, y analizar su asociación con los niveles de PTH.

## Materiales y métodos

### Pacientes y diseño del estudio

Se realizó un estudio observacional histórico transversal. La población del estudio estaba constituida por pacientes con peticiones de análisis de 25-(OH)D entre el 1 de enero y el 31 de diciembre de 2018, registradas en la base de datos del sistema informático del laboratorio (LIMS, Openlab 10.0.43, Nexus). Así mismo, se recogieron otros datos de interés, esto es, PTH, calcio (en suero y orina), fosfato (en suero y orina), creatinina (en suero y orina), magnesio sérico, fosfatasa alcalina total, gamma-glutamiltransferasa, lactato deshidrogenasa, bilirrubina total y glutamato-piruvato deshidrogenasa.

**Figura 1:**
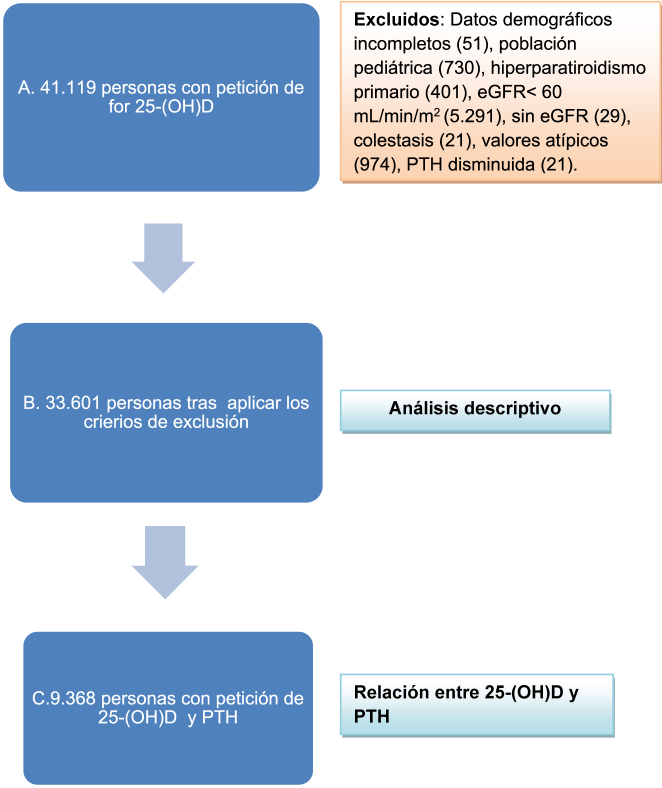
Participantes del estudio. Se especifican los criterios de exclusión, así como los análisis realizados en cada grupo. (A) Personas con petición de 25-(OH) D, (B) tras aplicar los criterios de exclusión, y (C) con petición conjunta de 25-(OH) D y PTH. 25-(OH) D, 25-hidroxivitamina D; eGFR, tasa de filtración glomerular estimada; PTH, hormona paratiroidea.

Se excluyeron las peticiones donde no figurara la fecha de nacimiento, las muestras de población pediátrica (<18 años), las muestras significativamente hemolizadas, las muestras de pacientes con insuficiencia renal crónica (IRC) estadio 3 ≥ (tasa de filtración glomerular estimada [eGFR] <60 mL/min/1,73 m^2^) [25]; pacientes con hiperparatiroidismo primario (PTH>65 pg/mL y calcio>10,3 mg/dL) o hipoparatiroidismo (PTH<12 pg/mL) (intervalo de referencia de la PTH 12–65 pg/mL), así como a aquellos pacientes con enfermedad hepática o colestasis. Del mismo modo, se descartaron los valores atípicos (e.g. valores con un valor z inferior a −3 o superior a 3) para los principales parámetros, esto es, 25-(OH)D, PTH, y calcio (Figura 1A–C). En el caso de los pacientes con más de una petición, solo se incluyó la primera cuantificación.

### Materiales

Se midieron una serie de parámetros en un analizador ARCHITECT c16000 (Abbott) mediante espectrofotometría, por medio de complejos coloreados o reacciones enzimáticas: calcio sérico y urinario, creatinina sérica y urinaria, fosforo sérico y urinario, magnesio sérico, fosfatasa alcalina total, gamma-glutamiltransferasa, bilirrubina total, lactato deshidrogenasa y glutamato-piruvato transaminasa. La PTH se midió mediante electroquimioluminiscencia (PTH intacta, STAT [tiempo corto de respuesta]) en un analizador COBAS e411. La 25-(OH)D se determinó mediante inmunoanálisis de micropartículas quimioluminiscente (CMIA) en el analizador ARCHITECT i2000sr (Abbott). Este parámetro ha sido estandarizado con respecto al método de referencia (cromatografía líquida de alta resolución: espectrometría de masas (LC-MS) basada en el Programa de Acreditación y Estandarización de la Vitamina D del Centro de Control de Enfermedades y Prevención (VDSCP CDC, ListNumber 5P02). Todas las muestras se recogieron siguiendo las especificaciones técnicas del fabricante. Se utilizaron tubos EDTA, con el fin de preservar la estabilidad de la PTH.

### Análisis estadístico

Se incluyó a un total de 33.601 personas (9.573 hombres; 24.028 mujeres) en el análisis descriptivo basado en los resultados disponibles (Figura 1). Entre las medidas estadísticas empleadas se incluyeron la media $\left(\bar{\text{X}}\right)$ y la desviación estándar (DE). La prevalencia del déficit de vitamina D se calculó según el sexo, edad y estación del año, tomando como referencia un punto de corte local para la 25-(OH)D de <20 ng/mL.

Las diferencias entre las medias de las variables cuantitativas entre los dos grupos se evaluaron mediante la prueba *t* de Student. Las diferencias entre los valores medios de 25-(OH)D entre las estaciones del año se evaluaron mediante análisis de varianza (ANOVA) y la prueba post hoc de Scheffé. Este análisis también se realizó en aquellos pacientes en los que se analizó la 25-(OH)D durante las útlimas dos semanas de cada estación, ya que las variaciones durante este periodo son más representativas del efecto de la estación en los niveles séricos de 25-(OH)D.

La relación entre la 25-(OH)D y la PTH se evaluó mediante análisis de regresión en una población de 9.368 sujetos (Figura 1). La primera fase consistió en un análisis de regresión lineal. El estudio se completó con un análisis de regresión logística multivariante entre la PTH y las variables predictivas 25-(OH)D, sexo, edad y estación. Nuestro análisis se basó en un modelo completo en el que se incluyeron todos los predictores de interés, la 25-(OH)D (edad, sexo, estación) y la interacción entre dichos predictores y la propia 25-(OH)D. Las variables se fueron eliminando una a una, comenzando por aquellas con los valores menos significativos (valor p elevado). Se repitió el ciclo con el modelo cada vez que se eliminaba una variable hasta obtener el modelo final, esto es, uno que únicamente contuviera las variables estadísticamente significativas y/o las variables con un efecto de confusión sobre la 25-(OH)D (se definió “confusión” como una odds ratio (OR) para la 25-(OH)D que se modificara en más del 10% cuando se eliminaba una variable). La bondad de ajuste se evaluó mediante la prueba de Hosmer-Lemeshow. La capacidad de discriminación del modelo se analizó mediante el área bajo la curva (AUC). El nivel de significación estadística se determinó en un valor p<0,05 (bilateral). La precisión de las estimaciones se basó en un intervalo de confianza del 95% (IC95%). El análisis estadístico se realizó con el programa SPSS Versión 15.0 (SPSS Inc., Chicago, Illinois, EE.UU).

## Resultados

En la Tabla 1 se muestran las estadísticas descriptivas para los parámetros analizados en la población de estudio. La edad media de la población de estudio fue de 59 años (58 en los hombres y 60 en las mujeres). Se observaron diferencias entre los niveles medios de PTH en función del sexo, con valores por encima del intervalo de referencia para los individuos sanos (hombres: 65,4 pg/mL; mujeres: 70,4 pg/mL), así como en los niveles medios de 25-(OH)D (hombres: 20,94 ng/mL; mujeres: 22,75 ng/mL). Se observaron diferencias estadísticamente significativas entre hombres y mujeres en los niveles de PTH y 25-(OH)D (p<0.001).

**Tabla 1:** Distribución de los parámetros analizados en la población de estudio.

	$\bar{\text{X}}$	DE	Mínimo	Máximo
Edad, años	59	18	18	106
25-(OH)D, ng/mL	22	12	1	67
PTH, pg/mL	69	30	12	204
Calcio, mg/dL	9,41	0,39	8,2	10,6
Fósforo, mg/dL	3,32	0,53	1,1	6,7
Magnesio, mg/dL	1,98	0,2	1	3
Calcio/creatinina	0,16	0,09	0	0,8
Reabsorción tubular de fosfato, %	82	7,2	17	97

PTH, hormona paratiroidea; DE, desviación estándar.

Los datos sobre la prevalencia del déficit de 25-(OH)D según el sexo indican que dicho problema era más prevalente entre los hombres (53% [5.030/9.573]) que en las mujeres (46%[11.103/24.028]). La prevalencia global fue del 48% (16.133/33.601).

La prevalencia del déficit de 25-(OH)D según el sexo y la estación del año se muestra en la Tabla 2. Por grupos de edad (Q1–Q4), la prevalencia fue mayor en el cuartil 1 (Q1, 74–87 años) y menor en el cuartil 2 (Q2, 60–73 años). Con respecto a la estación del año, se observó una mayor prevalencia del déficit de vitamina D en la primavera, siendo menor en verano. La diferencia interestacional media entre la primavera y el verano fue de 4,07 ng/mL (Tabla 3). Las diferencias entre los valores medios de 25-(OH)D entre las estaciones del año fueron significativas en ANOVA, excepto para la diferencia entre la primavera y el invierno. Cuando analizamos la 25-(OH)D durante las últimas dos semanas de cada estación, se observaron niveles inferiores de 25-(OH)D en el otoño. De nuevo, las diferencias entre medias fueron significativas, excepto para las diferencias entre la primavera y el invierno.

**Tabla 2:** Prevalencia del déficit de 25-(OH)D por cuartil de edad y estación del año.

Cuartil de edad		Invierno		Otoño		Primavera		Verano	
		n	%	n	%	n	%	n	%
Q1	Déficit	1.257	54	1.026	52	1.427	56	763	51
	(IC95%)		(52–56)		(50–54)		(54–58)		(49–53)
	Total	2.343		1.968		2.569		1.486	
Q2	Déficit	1.144	46	789	37	1.221	49	487	33
	(IC95%)		(44–48)		(35–39)		(47–51)		(31–35)
	Total	2.467		2.120		2.482		1.497	
Q3	Déficit	1.248	53	775	43	1.288	54	510	32
	(IC95%)		(51–55)		(41–45)		(52–56)		(30–34)
	Total	2.345		1.786		2.394		1.576	
Q4	Déficit	1.490	61	861	42	1.385	60	462	26
	(IC95%)		(59–63)		(40–44)		(58–62)		(24–28)
	Total	2.437		2.048		2.311		1.772	
	Déficit	5.139	54	3.451	44	5.321	55	2.222	35
	(IC95%)		(52–56)		(42–46)		(53–57)		(33–37)
	Total	9.592		7.922		9.756		6.331	

Cuartiles de edad: Q1 (74–87 años), Q2 (60–73 años), Q3 (46–59 años), Q4 (32–45 años); Déficit, Déficit de 25-(OH)D.

**Tabla 3:** Concentraciones séricas de 25-(OH)D según la estación del año.

Estación	Primavera	Invierno	Otoño	Verano
$\bar{\text{X}}$ , ng/mL	20,95	21,09	22,95	25,04
DE	12,36	12,37	11,98	12,19

DE, desviación estándar.

El análisis de regresión lineal de la vitamina D y la PTH reveló una relación débil, con un coeficiente de −0,303 (IC95%, entre −0,349 y −0,257; p<0,001).

La relación entre la PTH y las variables predictivas de 25-(OH)D, sexo, edad y estación del año, se evaluó mediante un análisis de regresión logística multivariante. Con el fin de descartar interacciones entre las variables predictivas, se construyó un modelo inicial en el que se incluyeron todas las interacciones posibles entre las variables independientes y la variable principal (25-(OH)D). En el modelo, se incluyeron las variables para las que no se observó interacción y las variables con interacción doble, tripe o cuádruple con la 25-(OH)D. Las interacciones no significativas se eliminaron jerárquicamente hasta obtener un modelo final (Tabla 4).

**Tabla 4:** Análisis de regresión logística (frente a PTH).

	Coeficiente	OR ajustada	IC 95%		Valor de p
	Beta		Inferior	Superior	
25-(OH)D	0,49	1,63	1,37	1,94	<0,001
Sexo femenino	0,24	1,27	1,16	1,39	<0,001
Estaciones					<0,001
Primavera	0,15	1,16	1,02	1,32	0,02
Invierno	0,49	1,63	1,44	1,86	<0,001
Otoño	0,1	1,11	0,96	1,28	0,16
Cuartiles edad (Q1–Q3)					<0,001
Q1	1,1	3	2,51	3,59	<0,001
Q2	1,08	2,96	2,51	3,49	<0,001
Q3	0,63	1,88	1,59	2,21	<0,001
Cuartil edad* 25-(OH)D					0,002
Q1*25-(OH)D	0,35	1,42	1,09	1,84	0,009
Q2* 25-(OH)D	−0,1	0,91	0,71	1,16	0,44
Q3*25-(OH)D	−0,1	0,9	0,71	1,14	0,4
Constante	−1,18	0,31			<0,001

Cuartiles de edad (Q1–Q3): Q1 (74–87 años), Q2 (60–73 años), Q3 (46–59 años), Q4 (32–45 años). OR, odds ratio; PTH, hormona paratiroidea.

## Discusión

La prevalencia del déficit de 25-(OH)D difería según el sexo, la edad y la estación del año. Además, el análisis multivariante mostró que la edad, la estación del año y el sexo influían en la relación entre la 25-(OH)D y la PTH. Aportamos resultados de prevalencia del déficit de 25-(OH)D en una amplia serie de individuos.

La prevalencia del déficit de vitamina D en nuestro entorno es de alrededor del 48%, un porcentaje similar al referido en Nueva Zelanda [26]. Existen diferentes estudios de series sobre la prevalencia del déficit de 25-(OH)D en España en diferentes grupos de edad (población pediátrica, mujeres posmenopáusicas, personas de edad avanzada), independientemente del nivel de exposición a la luz solar [27], [28], [29], [30], [31], [32].

No existe una prevalencia estimada homogénea del déficit de 25-(OH)D en España. Los estudios incluyen poblaciones muy dispares (personas de edad avanzada, mujeres posmenopáusicas y población pediátrica), existiendo notables diferencias en los métodos de cuantificación de la 25-(OH)D (radioinmunoensayo, ensayos de unión de ligandos, HPLC), lo que impide un estudio comparativo entre las personas afectadas.

En su estudio sobre la prevalencia de la hipovitaminosis D en Madrid, Aguado y col. [27] reportan una prevalencia del 84% del déficit en 171 mujeres posmenopáusicas con enfermedad reumática, basándose en un valor de referencia de 20 ng/mL. La 25-(OH)D se midió mediante radioinmunoensayo. Mata-Granados y col. [29] revelan una prevalencia del déficit de vitamina D del 51% (25-(OH)D entre 10 y 20 ng/mL) en hombres y mujeres de entre 18 y 65 años en Córdoba, donde la 25-(OH)D se determinó mediante HPLC. Quesada y col. [28] informan de una prevalencia del déficit de la 25-(OH)D del 44% en mujeres posmenopáusicas no tratadas y del 29% en mujeres tratadas. La edad media fue de 71 años, y la muestra estaba compuesta por mujeres de toda España (28–43°N). La 25-(OH)D se midió mediante HPLC. Lips y col. [30] informan de una prevalencia del 42% en mujeres de 64 años con osteoporosis (37–43°N), tras determinar la 25-(OH)D mediante radioinmunoensayo.

En Europa, Cashman y col. [19] registraron valores séricos de 25-(OH)D estandarizados mediante VDSP (cuantificación mediante HPLC-MS). El análisis de un total de 55.844 muestras de todas las edades procedentes de 14 países arrojó una prevalencia del 40% (valor de referencia: 20 ng/mL).

En nuestra serie, la prevalencia del déficit fue mayor en los hombres que en las mujeres (53% vs. 46%). Coincidiendo con nuestro estudio, AlQuaiz y col. [33] documentaron una prevalencia superior entre los hombres, en un estudio retrospectivo de personas de entre 30 y 75 años (n=2.835 pacientes) en atención primaria en Arabia Saudí.

Las diferencias entre sexo observadas en nuestro estudio coinciden con los resultados de un estudio sobre la prevalencia del déficit de 25-(OH)D en Chile [34] (latitud 33°S). Los autores calcularon la prevalencia del déficit de 25-(OH)D por sexos en personas sanas de entre 18 y 89 años (n=1.329) y obtuvieron valores superiores en los hombres (45.9%). Además, las diferencias entre hombres y mujeres se atribuyeron a una menor exposición a la luz solar por parte de los hombres frente a las mujeres, principalmente a las mujeres jóvenes. Los principales factores que subyacen en dicha discrepancia son el mayor porcentaje de labores fuera del hogar y a un estilo de vida sedentario entre los hombres.

Entre las mujeres, la menopausia podría ser un factor a tener en cuenta a la hora de evaluar el déficit de 25-(OH)D. Según la literatura científica, la relación entre la menopausia y la hipovitaminosis D no ha mostrado una relación causal, sino una asociación meramente epidemiológica.

Por un lado, la determinación de los niveles de vitamina D es habitual en las mujeres posmenopáusicas, al menos en un primer examen. Sin embargo, la determinación en otros contextos clínicos es muy poco frecuente en los países desarrollados. Así, la relación entre la hipovitaminosis D en las mujeres posmenopáusicas es relativamente frecuente, en primer lugar porque a este grupo de población se le suele realizar este análisis con mayor frecuencia.

El tiempo de exposición a la luz solar también disminuye con la edad, ya que se va adoptando un estilo de vida más sedentario. Este hecho podría también explicar la relación entre menopausia e hipovitaminosis D. Otro factor intermediario conocido entre la menopausia y el déficit de vitamina D es la obesidad central y los síndromes de malabsorción. Estas patologías también son más frecuentes en personas de edad y, por lo tanto, en la población posmenopáusica [7, 35, 36].

Con respecto a la estación del año, en nuestro estudio, este déficit mostró una mayor prevalencia en primavera (diferencias no significativas con respecto al invierno) y menor en verano. La presencia de variaciones interestacionales también se confirmó cuando se repitió la comparación entre personas en las que se cuantificó la 25-(OH)D en las últimas dos semanas de cada estación del año. En este caso, el déficit de 25-(OH)D fue menor en verano y mayor hacia finales del otoño, lo que podría explicar los niveles inferiores de 25-(OH)D durante los meses de invierno. Postulamos que las variaciones en las concentraciones séricas de 25-(OH)D entre el final del verano y el principio del otoño se podrían deber a una mayor exposición a la luz solar en verano y a la falta de suplementación durante estos meses.

Nuestro estudio confirma las variaciones estacionales de 25-(OH)D a lo largo del año documentadas anteriormente. Los resultados obtenidos en este estudio coinciden con los de estudios anteriores [37], [38], [39], lo que demuestra una mayor prevalencia del déficit de 25-(OH)D en invierno y primavera. Las principales discrepancias en nuestro estudio se observaron durante la estación con la menor prevalencia del déficit (verano, frente al otoño en los estudios citados anteriormente).

La síntesis cutánea de 25-(OH)D disminuye durante el invierno, debido a la menor exposición a la luz solar, en comparación con el verano. La latitud también desempeña un papel fundamental, ya que existen diferencias notables entre las regiones que se encuentran por encima y por debajo de la latitud 37°N. De este modo, las personas que habitan por encima de esta latitud tienen menos posibilidad de realizar la síntesis cutánea de 25-(OH)D durante los meses de invierno, debido al ángulo de incidencia de los rayos UV [40]. Nuestra región tiene latitud 40°N, lo que debe ser tenido en cuenta a la hora de evaluar los resultados.

Pereda y col. [37] hallaron déficit de vitamina D en mujeres adultas con enfermedad reumática (media, 53,2 años) en la región de Almería (España) (latitud 36°N). En una población total de 319 pacientes (81,5% posmenopáusicas) que no tomaban suplementos de 25-(OH)D, los niveles medios de vitamina D fueron de <30 ng/mL, excepto en otoño, a pesar de la óptima radiación solar.

Gozdzik y col. [38] documentaron una variación interestacional en el déficit de 25-(OH)D en adultos jóvenes (18–35 años) en Toronto (Canadá) (latitud 43°N). Los autores determinaron los niveles de 25-(OH)D en otoño e invierno durante dos años y observaron diferencias medias de 6 ng/mL entre estas dos estaciones (21,8 y 15,8, respectivamente). Las diferencias interestacionales en su población de estudio dependió principalmente de la toma de suplementos de vitamina D y la pigmentación de la piel.

En términos de edad, la mayor prevalencia del déficit de 25-(OH)D se observó en los pacientes de edad más avanzada (Q1: 74–87 años). Esto coincide con dos estudios realizados en España, que muestran una elevada prevalencia en los pacientes de mayor edad [31, 32]. En ambos estudios, la prevalencia fue del 87% en las personas mayores institucionalizadas [31] y no institucionalizadas [32] en Barcelona.

Tal como indica la literatura científica [20, 21], se utilizó la PTH como variable de resultado principal debido a su asociación clínica con la 25-(OH)D, aunque existe una correlación débil entre estos dos parámetros. Nuestro estudio confirma dicha correlación (coeficiente: −0,303).

El análisis multivariante reveló una relación estadísticamente significativa entre el déficit de 25-(OH)D y niveles de PTH elevados (OR, 1,63). Otros factores de riesgo asociados a los niveles elevados de PTH incluyen el sexo femenino (OR, 1,27), la estación del año (invierno, OR 1,63, primavera OR 1,16) y la edad (Q1, OR, 3).

El efecto del sexo femenino sobre la PTH se observa en el hecho de que los niveles plasmáticos de PTH son mayores en las mujeres que en los hombres (70,4 pg/mL y 65,4 pg/mL, respectivamente). Dichas diferencias en la PTH entre hombres y mujeres ya han sido documentadas en estudios anteriores [41].

El estudio de las interacciones entre variables predictivas (estación, sexo, edad) y la PTH descartó todas las interacciones, excepto la de la edad con la 25-(OH)D (p<0,05); razón por la cual éstas se excluyeron del modelo final. Con respecto a los grupos de edad, la única interacción observada con la 25-(OH)D fue la de la edad avanzada (Q1) (p=0,009). Este resultado sugiere un posible efecto de la edad sobre los niveles séricos de 25-(OH)D, por lo que la edad debería ser tenida en cuenta a la hora de evaluar la relación entre la 25-(OH)D y la PTH. Las personas de edad avanzada presentan un mayor riesgo de tener niveles elevados de PTH y exhiben niveles séricos de 25-(OH)D inferiores a los del resto de la población. Cuando la edad y la 25-(OH)D se observan juntos únicamente en personas mayores con déficit de 25-(OH)D, la PTH aumenta.

Sorprendentemente, el efecto de la edad sobre la 25-(OH)D y de la 25-(OH)D en la PTH no se observa en otros grupos de personas de edad avanzada, tales como aquellas del grupo Q2 (60–73 años). De hecho, la menor prevalencia observada del déficit de 25-(OH)D se documentó en este cuartil, probablemente debido a que este grupo de población suele tomar suplementos, a diferencia de la población de otros cuartiles. No obstante, no pudimos demostrar esta aseveración.

Con respecto a la estación del año, la elevación de los niveles de PTH estuvo principalmente asociada al invierno y la primavera (el otoño no fue significativo), lo que indica estacionalidad, tal como observamos en la 25-(OH)D. Esta variación interestacional de la PTH ya ha sido documentada anteriormente [42]. De este modo, la estacionalidad debe ser tenida en cuenta a la hora de evaluar los valores de 25-(OH)D y PTH.

El modelo estaba correctamente calibrado, tal como se confirmó mediante la prueba de Hosmer-Lemeshow (p=0,842). El AUC fue de 0,656, cerca del valor de corte aceptable (AUC ≥0,7), lo que indica un bajo grado de discriminación del modelo entre pacientes con valores de PTH normales y aquellos con valores superiores a los valores de referencia. Este resultado confirma que la 25-(OH)D como variable predictiva clave no puede explicar un gran porcentaje de las variaciones en la PTH (34,4% en nuestra serie).

Nuestro estudio presenta varias fortalezas. En primer lugar, el tamaño de la muestra poblacional, lo que implica que nuestro estudio tiene una considerable potencia estadística. En segundo lugar, empleamos una técnica de cuantificación de 25-(OH)D sólida, ya que ésta ha sido estandarizada por el CDC. Esto garantiza un error sistemático de ±5% y una imprecisión de <10% con respecto al método de referencia para la determinación de este analito.

La principal limitación de nuestro estudio es su diseño histórico. Puede existir un sesgo en la selección de pacientes, ya que la prevalencia del déficit de 25-(OH)D se calculó a partir de datos de pacientes a los que se sometió a la prueba; así mismo su asociación con la PTH se evaluó en pacientes a los que se realizó una prueba conjunta de 25-(OH)D y PTH. En un estudio ideal, tanto la 25-(OH)D como la PTH se deberían determinar en todos los pacientes. Además, el diseño del estudio no nos permitió distinguir entre los pacientes que tomaban suplementos y los que no.
